# Right Ventricular Morphology and the Onset of Dyspnea: The MESA-Right Ventricle Study

**DOI:** 10.1371/journal.pone.0056826

**Published:** 2013-02-15

**Authors:** Michael R. Kaufmann, R. Graham Barr, João A. C. Lima, Amy Praestgaard, Aditya Jain, Harikrishna Tandri, David A. Bluemke, Steven M. Kawut

**Affiliations:** 1 Department of Medicine, University of Pennsylvania, Philadelphia, Pennsylvania, United States of America; 2 Penn Cardiovascular Institute, University of Pennsylvania, Philadelphia, Pennsylvania, United States of America; 3 Department of Medicine, College of Physicians and Surgeons, Columbia University, New York, New York, United States of America; 4 Department of Epidemiology, Mailman School of Public Health, Columbia University, New York, New York, United States of America; 5 Department of Medicine, Johns Hopkins University School of Medicine, Baltimore, Maryland, United States of America; 6 Center for Clinical Epidemiology and Biostatistics, Perelman School of Medicine, University of Pennsylvania, Philadelphia, Pennsylvania, United States of America; 7 Radiology and Imaging Sciences NIH Clinical Center, National Institute of Biomedical Imaging and Bioengineering, Bethesda, Maryland, United States of America; Indiana University, United States of America

## Abstract

**Background:**

The association of right ventricular (RV) structure and function with symptoms in individuals without cardiopulmonary disease is unknown. We hypothesized that greater RV mass and RV end-diastolic volume (RVEDV), smaller RV stroke volume (RVSV), and lower RV ejection fraction (RVEF) measured by cardiac magnetic resonance imaging (MRI) in participants free of clinical cardiovascular disease at baseline would be associated with a greater risk of self-reported dyspnea.

**Methods:**

The Multi-Ethnic Study of Atherosclerosis (MESA) performed cardiac MRIs on participants without clinical cardiovascular disease between 2000 and 2002. We excluded subjects who reported “prevalent” dyspnea at the first assessment (24 months). The presence of dyspnea was assessed at 24 months, 42 months, and 60 months from baseline. Cox proportional hazards models were used to examine the relationship between RV measures and incident dyspnea.

**Results:**

In the final study sample (N = 2763), there were significant interactions between RV measures and sex in terms of the risk of dyspnea (p<0.05). Among men (N = 1453), lower RV mass (p = 0.003), smaller RVEDV (p<0.001), smaller RV end-systolic volume (RVESV) (p = 0.03) and decreased RVSV (p<0.001) were associated with an increased risk of developing dyspnea after adjusting for covariates. Associations remained after adjusting for left ventricular function and lung function. However, there were no significant associations between RV measures and the risk of dyspnea in women.

**Conclusions:**

Lower RV mass and smaller RV volumes were associated with an increased risk of dyspnea in men, but not in women.

## Introduction

While the importance of changes in left ventricular (LV) structure and function is well-defined, our understanding of the consequences of structural and functional changes in the RV is quite limited by comparison. As two pumps in series, the LV cardiac output (and systemic perfusion) is dependent on RV function in both healthy individuals and those with disease. Therefore, subclinical changes in RV morphology could herald the onset of cardiopulmonary limitation, prior to detectable LV changes or symptoms.

RV structure and function are associated with outcomes in patients with cardiovascular disease [1], [2], [3], [4]. However, there are no studies of the association of RV morphology with symptoms in individuals without clinical cardiovascular disease. An association of RV parameters in individuals without known cardiovascular disease with subsequent activity-limiting dyspnea could reflect either a direct contribution of RV function to functional status or the impact of sub-clinical lung disease on the RV. In either case, RV morphology could be an important subclinical indicator of cardiopulmonary dysfunction. We hypothesized that greater RV end-diastolic mass (RVEDM), larger RV end-diastolic volume (RVEDV), smaller RV stroke volume (RVSV), and lower RV ejection fraction (RVEF) at baseline, due to increased RV afterload from underlying lung or heart disease, would be associated with a greater risk of the development of self-reported dyspnea.

## Methods

### The Multi-Ethnic Study of Atherosclerosis

The Multi-Ethnic Study of Atherosclerosis (MESA) is a multicenter prospective cohort study to investigate the prevalence, correlates and progression of subclinical cardiovascular disease in whites, African Americans, Hispanics, and Chinese [5]. In 2000–2002, MESA recruited 6,814 men and women aged 45–84 years old from six U.S. communities: Forsyth County, NC; Northern Manhattan and the Bronx, NY; Baltimore City and Baltimore County, MD; St. Paul, MN; Chicago, IL; and Los Angeles, CA. Exclusion criteria included clinical cardiovascular disease (physician diagnosis of heart attack, stroke, transient ischemic attack, heart failure, angina, current atrial fibrillation, any cardiovascular procedure), weight >136 kg (300 lbs), pregnancy, or impediment to long-term participation. The protocols of MESA and all studies described herein were approved by the Institutional Review Boards of all collaborating institutions (Columbia University, New York; Johns Hopkins University, Baltimore; Northwestern University, Chicago; University of California, Los Angeles; University of Minnesota, Twin Cities; Wake Forrest University, Winston-Salem) and the National Heart Lung and Blood Institute. Written informed consent was obtained from all participants.

**Figure 1:**
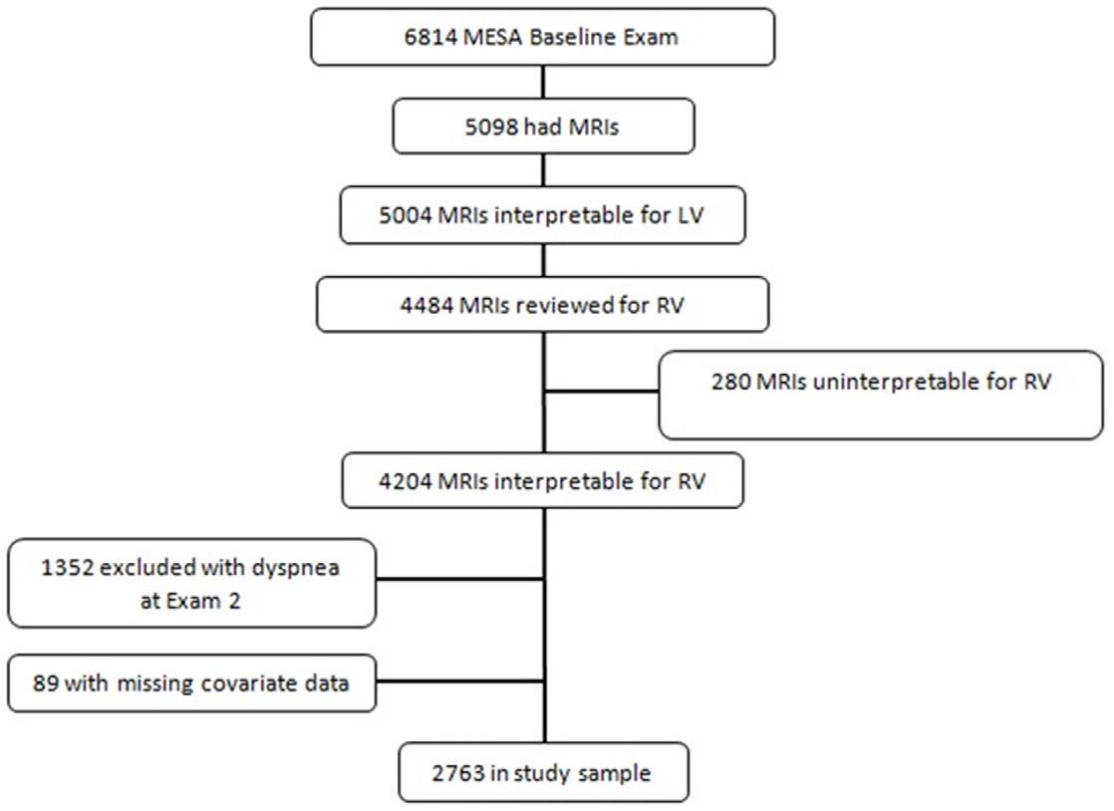
Study sample.

The MESA-Right Ventricle Study is an ancillary study supported by an NIH grant that planned for the interpretation of approximately 4200 cardiac MRIs at the baseline examination (Exam 1) for measurement of RV morphology. We selected 4634 scans for RV interpretation, and 4484 had interpretation attempted. We successfully interpreted scans of 4204 individuals (94% of attempted). Participants were sampled without regard to demographics, anthropometrics, or other clinical variables. Since questionnaire data regarding dyspnea were not collected at Exam 1, we included only those subjects who denied dyspnea at Exam 2 to exclude those with prevalent dyspnea (see below) (Figure 1).

### Cardiac Magnetic Resonance Imaging Measures

The cardiac MRI protocol has been previously described [6]. All imaging was performed on 1.5 T magnets with a 4-element phased-array surface coil positioned anteriorly and posteriorly and electrocardiographic gating. Imaging consisted of fast gradient echo cine images with temporal resolution ≤50 ms.

Methods for interpretation of LV and RV parameters have been previously reported [7], [8]. Briefly, RV image analysis was performed by two independent analysts on Windows workstations using QMASS software (v4.2, Medis, the Netherlands). The endocardial and epicardial borders of the RV were traced manually on the short axis cine images at the end-systolic and end-diastolic phase. RV end-systolic volume (RVESV) and RVEDV were calculated using Simpson's rule by summation of areas on each slice multiplied by the sum of slice thickness and image gap. RV mass was determined at the end-diastole phase as the difference between end-diastolic epicardial and endocardial volumes multiplied by the specific gravity of the heart (1.05 g/cm^3^). RVSV was calculated by subtracting RVESV from the RVEDV. RVEF was calculated by dividing RVSV by RVEDV. The intra-reader intraclass coefficients (ICCs) for random, blinded re-reads of 230 scans were 0.99 for RVEDV, 0.89 for RVEF and 0.94 for RV mass (N = 229). The inter-reader ICCs from random, blinded re-reads of 240 scans for RV mass and RVEDV were 0.89 and 0.96 respectively and 0.80 for RVEF.

### Dyspnea

The MESA medical history questionnaire was administered to participants at Exams 2, 3, and 4 (approximately 24 months, 42 months, and 60 months, respectively, after the baseline exam and cardiac imaging) and contained the following questions: “When walking on level ground, do you get more breathless than people your own age?” “When walking up hills or stairs, do you get more breathless than people your own age?” “Do you ever have to stop walking because of breathlessness?” The answer to each of these questions was assessed independently. Time from Exam 2 to an affirmative answer to any one of these three questions at Exam 3 or 4 (or death) was considered as the dependent variable. Death was included because this event was likely informative, defying the assumptions required for censoring in survival analysis.

### Covariates

Race/ethnicity was self-reported during the baseline MESA exam according to 2000 US Census criteria as race (Caucasian, African-American, Chinese) and ethnicity (Hispanic or non-Hispanic). Participants self-identifying as Hispanic were categorized as Hispanic. Standard questionnaires were used to ascertain smoking status (classified as never, former, or current) and pack years. Resting blood pressure was measured three times using the Dinamap Monitor PRO 100 (Critikon, Tampa, FL) automated oscillometric device, and the average of the last two measurements was used. Hypertension was defined as systolic blood pressure ≥140 mmHg, diastolic blood pressure ≥90 mmHg or self-reported hypertension and current use of anti-hypertensive medication. Presence of diabetes mellitus was based on self-reported physician diagnosis, use of medication for hyperglycemia, or a fasting glucose value ≥126 mg/dL, the latter measured by rate reflectance spectrophotometry (Johnson & Johnson Clinical Diagnostics, Inc., Rochester, NY). Fasting glucose between 100–125 mg/dL was considered impaired fasting glucose. Fasting blood samples were drawn and sent to a central laboratory for measurement of glucose and lipids. Total cholesterol was assessed using standard methods. Serum creatinine was measured by rate reflectance spectrophotometry (Johnson & Johnson Clinical Diagnostics, Inc., Rochester, NY 14650). IL-6 was measured by ultra-sensitive ELISA (Quantikine HS Human IL-6 Immunoassay; R&D Systems, Minneapolis, MN). C-reactive protein (CRP) was measured using the BNII nephelometer (N High Sensitivity CRP; Dade Behring Inc., Deerfield, IL). Insulin was determined by a radioimmunoassay method using the Linco Human Insulin Specific RIA Kit (Linco Research, Inc., St. Charles, MO 63304).

Of the patients with available RV measures from Exam 1 (2000–2002), there were 1969 patients with available additional measures of spirometry (forced expiratory volume in 1 sec (FEV1), forced vital capacity (FVC)), urine cotinine, and percent emphysema measured on the lung windows of cardiac computed tomography (CT) performed in Exam 2 (2004–2006).These subjects (part of another ancillary study “MESA-Lung”) were not selected based on RV morphology or other characteristics. Details of these measures are published [9], [10].

### Statistical Analysis

Continuous variables were expressed as means and standard deviations or ranges. Categorical variables were expressed as %. We used survival analysis to characterize the relationship between RV parameters at baseline (independent variables) and time to an exam when dyspnea was reported (or death) (dependent variable). To account for the interval censoring inherent in using the exam questionnaires, the time variable was coded as 1 or 2 to correspond to the time from Exam 2 to Exams 3 or 4 respectively. Deaths were assigned to the next exam time variable (i.e. a death between Exams 3 and 4 was coded as a death at Exam 4), to minimize differential misclassification of time between the dyspnea outcome and death.

Bivariate and multivariate Cox proportional hazards models were estimated with RV parameters as continuous variables. Potential covariates were assessed based on known associations with ventricular size and heart disease, including demographics and anthropometric variables, as well as variables reflecting comorbidities and other characteristics (including systolic and diastolic blood pressure, hypertension, diabetes mellitus, history of venous thromboembolism, self-reported intentional exercise, use of anti-hypertensives, aspirin, statins, lipid-lowering therapy, hormone replacement therapy, HgA1c, total cholesterol, low- and high-density lipoproteins, triglycerides, fasting glucose and insulin levels, serum IL-6, C-reactive protein (CRP), serum creatinine). Covariates which were measured at each exam (anthropometrics, diabetes status, systolic and diastolic blood pressure, total cholesterol, HDL, LDL, triglycerides, creatinine, glucose, CRP, total intentional exercise, smoking status, hypertension, use of anti-hypertensives, and others) were assessed as time-varying covariates. We retained covariates in all of the models which changed the coefficient of the RV parameter by >15% in any model. Retained covariates included age, race/ethnicity, education, body mass index, waist size, hip size, smoking status, diastolic blood pressure, hypertension, creatinine, and IL-6. We forced education level into the models to account for differences in socioeconomic status, as recommended by the MESA parent study. Adjusting for body mass index, waist size and hip size serves to index RV measures to body size, so that additional indexing (e.g., body surface area) was not necessary.

Additional models adjusted for all of the confounders plus the respective LV measure. Adjustment for LV parameters was performed to focus specifically on the RV, rather than bi-ventricular processes. RVSV was not adjusted for LV stroke volume (LVSV) considering the significant inter-dependence of these measures. The LVSV is wholly dependent on the RVSV, so it cannot be a confounder. Selected interactions between demographics and RV parameters, and RV and LV parameters, were assessed.

Primary analyses were performed with all participants with complete data for covariates. Subset analyses were performed in participants with available pulmonary measures from the MESA-Lung Study. Sensitivity analyses were performed considering death as a censoring point. P values<0.05 were considered significant.

## Results

MESA included 6814 participants (Figure 1). Of these, 5098 had MRI performed, of which 5004 were interpretable for the LV. We selected 4634 scans for MESA-RV and attempted to interpret 4484, of which 4204 had interpretable RV measurements. The slightly lower reading success rate (94%) compared to the LV in MESA (98%) was likely attributable to the technical demands of interpreting the very thin RV free wall. Of those participants with interpretable RV measurements, 1352 participants were excluded because they reported dyspnea at Exam 2 (prevalent dyspnea). An additional 89 participants had incomplete covariate data, leaving a total of 2763 participants in the final study sample (and 4051excluded).

Table 1 shows the characteristics of the study sample compared to those excluded. The study sample had a mean age of 61.3±10.0 years, and 52.6% were men. Almost forty percent self-identified as Caucasian, 23.6% African-American, 21.6% Hispanic, and 15.0% Chinese. The mean BMI was 27.1±4.5 kg/m^2^. The study sample had slightly lower BMI, but was otherwise similar to those excluded. Formal statistical testing is not appropriate for such comparisons, which are descriptive rather than inferential. The mean RV mass in the study sample was 21.1±4.5 g, the mean RVEDV was 125.8±31.6 mL, the mean RVESV was 38.0±14.6 mL, and the mean RVSV was 87.7±20.8 mL. The mean RVEF was 70.3±6.4 %. All of these parameters were adjusted for body size in the multivariate analyses, so that further indexing (e.g., by body surface area) was unnecessary. Mean RV and LV measurements for the final study sample, stratified by gender, are shown in Table 2.

**Table 1:** Characteristics of study population.

Variable	Value	Male (N = 1453)	Female (N = 1310)	Study Sample (N = 2763)	Excluded (N = 4051)
Age	Mean +/− SD	61.1+/−10.0	61.5+/− 10.1	61.3+/−10.0	62.7+/−10.3
Sex	Male			1,453 (52.6)	1,760 (43.4)
Race	Caucasian	576 (39.6)	524 (40.0)	1,100 (39.8)	1,522 (37.6)
	Asian	212 (14.6)	202 (15.4)	414 (15.0)	389 (9.6)
	African American	339 (23.3)	313 (23.9)	652 (23.6)	1,241 (30.6)
	Hispanic	326 (22.4)	271 (20.7)	597 (21.6)	899 (22.2)
Education Level	< High School	201 (13.8)	217 (16.6)	418 (15.1)	807 (20.0)
	High School/GED	219 (15.1)	275 (21.0)	494 (17.9)	742 (18.4)
	< College (> High School)	364 (25.1)	376 (28.7)	740 (26.8)	1,197 (29.7)
	Bachelor's Degree	303 (20.9)	237 (18.1)	540 (19.5)	631 (15.7)
	Graduate Degree	366 (25.2)	205 (15.6)	571 (20.7)	651 (16.2)
BMI	Mean +/− SD	27.3+/−4.0	26.9+/−5.0	27.1+/−4.5	29.3+/−6.0
Waist Size (cm)	Mean +/− SD	96.7+/−11.1	92.2+/−13.6	94.6+/−12.6	100.3+/−15.4
Hip Size (cm)	Mean +/− SD	101.8+/−8.2	103.4+/−10.5	102.6+/−9.4	107.0+/−12.8
Total Intentional Exercise (MET-min per week)	Mean +/− SD	1743.2+/−2235.4	1379.6+/−2405.9	1570.8+/−2324.5	1270.9+/−1996.8
Diabetes mellitus	Normal (<100 mg/dl)	1,010 (69.5)	1,012 (77.3)	2,022 (73.2)	2,616 (64.7)
	Impaired Fasting Glucose (100–125 mg/dl)	248 (17.1)	172 (13.1)	420 (15.2)	721 (17.8)
	Untreated Diabetes (> = 126 mg/dl)	45 (3.1)	20 (1.5)	65 (2.4)	123 (3.0)
	Treated Diabetes	150 (10.3)	106 (8.1)	256 (9.3)	582 (14.4)
Fasting Glucose (mg/dL)– Calibrated	Mean +/− SD	100.3+/−27.2	94.1+/−21.5	97.4+/−24.9	102.5+/−34.0
Hypertension	Yes	589 (40.5)	536 (40.9)	1,125 (40.7)	2,053 (50.7)
Systolic Blood Pressure (mmHg)	Mean +/− SD	122.7+/−18.1	121.9+/−21.6	122.3+/−19.9	126.8+/−22.0
Diastolic Blood Pressure (mmHg)	Mean +/− SD	73.4+/−9.0	67.2+/−9.7	70.5+/−9.8	70.7+/−10.4
Use of Anti-Hypertensives	Yes	532 (36.6)	474 (36.2)	1,006 (36.4)	1,838 (45.4)
Total Cholesterol (mg/dl)	Mean +/− SD	185.4+/−34.0	199.3+/−33.7	191.9+/−34.5	191.4+/−36.9
HDL Cholesterol (mg/dl)	Mean +/− SD	46.7+/−12.3	58.6+/−16.0	52.3+/−15.3	51.3+/−14.9
LDL Cholesterol (mg/dl)	Mean +/− SD	112.9+/−31.4	115.9+/−30.7	114.3+/−31.1	113.6+/−33.1
Triglycerides (mg/dl)	Mean +/− SD	132.5+/−86.9	124.3+/−71.9	128.6+/−80.3	134.0+/−85.9
Statin Use	Yes	194 (13.4)	186 (14.2)	380 (13.8)	629 (15.5)
Creatinine (mg/dl)	Mean +/− SD	1.1+/−0.2	0.8+/−0.1	1.0+/−0.2	1.0+/−0.3
Interleukin-6 (IL-6) (pg/mL)	Mean +/− SD	1.3+/−1.1	1.4+/−1.1	1.4+/−1.1	1.7+/−1.3
Smoking Status	Never	582 (40.1)	802 (61.2)	1,384 (50.1)	1,788 (44.2)
	Former	730 (50.2)	392 (29.9)	1,122 (40.6)	1,712 (42.3)
	Current	141 (9.7)	116 (8.9)	257 (9.3)	546 (13.5)
Pack Years	Mean +/− SD	12.2+/−22.5	5.9+/−13.4	9.2+/−19.0	12.9+/−24.1

**Table 2:** Mean RV and LV measures stratified by gender.

Variable	Male (N = 1453)	Female (N = 1310)
RV EDM (g)	23.1+/−4.4	18.9+/−3.5
RV EDV (mL)	142.0+/−29.7	107.8+/−22.6
RV EF (%)	68.2+/−6.1	72.6+/−5.8
RV ESV (mL)	45.4+/−14.1	29.8+/−9.8
RV SV (mL)	96.5+/−20.5	78.0+/−16.3
LV EDM (g)	168.0+/−35.6	120.8+/−26.2
LV EDV (mL)	141.2+/−31.7	112.8+/−23.2
LV EF (%)	66.5+/−7.1	71.6+/−6.3
LV ESV (mL)	47.9+/−17.3	32.4+/−11.4

There were 12,659 person-years of follow-up, and the median follow-up was 4.74 years. The 5-year cumulative incidence of the report of dyspnea on hills or stairs was 13.6%, on level ground was 4.0%, and dyspnea causing the individual to stop was 5.6%. The 5-year risk of dyspnea of any type was 16.5%.

Lower RV mass was associated with an increased risk of dyspnea in the unadjusted model (Table 3). For every 1 SD (4 g) decrement in RV mass, there was a 15% increase in the risk of dyspnea or death. After adjustment for confounders, there was a 22% increase in the risk of dyspnea or death for men, but no association between RV mass and the risk of dyspnea or death for women (p for interaction = 0.006). Further adjustment for LV mass, if anything, strengthened the association in men (Table 3), suggesting that the association was RV-specific and independent of LV mass.

**Table 3:** Cox proportional hazards models for RV measures and time to dyspnea.

	HR*	95%CI	P value
**Right ventricle end-diastolic mass**			
Unadjusted model	1.15	1.06–1.24	<0.001
Adjusted model†			
Men	1.22	1.07–1.40	0.003
Women	0.95	0.83–1.09	0.49
Adjusted model + LV end-diastolic mass			
Men	1.33	1.15–1.54	<0.001
Women	1.02	0.88–1.17	0.82
**Right ventricle end-diastolic volume**			
Unadjusted model	1.12	1.06–1.18	<0.001
Adjusted model†			
Men	1.20	1.09–1.32	<0.001
Women	0.95	0.86–1.06	0.37
Adjusted model + LV end-diastolic volume			
Men	1.25	1.10–1.41	<0.001
Women	0.99	0.87–1.13	0.90
**Right ventricle ejection fraction**			
Unadjusted model	0.96	0.90–1.02	0.17
Adjusted model†	1.03	0.96–1.11	0.37
Adjusted model + LV ejection fraction	1.03	0.95–1.11	0.49
**Right ventricle end-systolic volume**			
Unadjusted model	1.24	1.10–1.40	<0.001
Adjusted model†			
Men	1.24	1.02–1.51	0.03
Women	0.85	0.67–1.07	0.16
Adjusted model + LV end-systolic volume			
Men	1.27	1.03–1.58	0.03
Women	0.87	0.68–1.11	0.25
**Right ventricle stroke volume**			
Unadjusted model	1.16	1.07–1.26	<0.001
Adjusted model†			
Men	1.30	1.14–1.49	<0.001
Women	0.96	0.83–1.11	0.56

* All hazard ratios correspond to ∼1 SD decrement in the respective RV measure (4 g for mass, 20 cc for volumes, 6% in RVEF).

† Adjusted model includes: age, race/ethnicity, education, body mass index, waist size, hip size, smoking status, diastolic blood pressure, hypertension, creatinine, and IL-6.

Smaller RVEDV, RVESV, and RVSV were also associated with significantly increased risks of dyspnea in unadjusted models (Table 3). One SD (20 mL) decrements in RV volumes were associated with 12–24% increases in the risk of dyspnea. After adjustment for confounders, there were 20–30% increases in the risk of dyspnea for men, but no association between RV volumes and the risk of dyspnea for women (p for interaction <0.001 for RVEDV,  = 0.011 for RVESV, and  = 0.001 for RVSV). For men, these associations persisted despite adjustment for confounders and after adjustment for the respective LV volumes. RVEF was not significantly associated with the risk of dyspnea (Table 3).

Subset analyses were performed in participants with available spirometry, urine cotinine, and chest CT (N = 1969). Participants had a mean FEV1 of 2487±728 ml, FVC of 3315±967 ml, and mean percent emphysema at a −910 Hounsfield Unit threshold of 17.4±12.0%. Mean spirometry, urine cotinine, and percent emphysema values, stratified by gender are shown in Table 4. Including age and body size measurements allowed the use of absolute spirometric measures in the adjusted analyses. In this subset, the associations of RV mass and RV volumes with the risk of dyspnea were similar to those from the full dataset (and stronger in most cases) (Table 5). Reclassifying death as a censoring point did not significantly affect the results (data not shown).

**Table 4:** Mean spirometry, urine cotinine, and percent emphysema values, stratified by gender.

Variable	Male (N = 1048)	Female(N = 921)
FEV1 (mL)	2893.7+/−656.6	2025.2+/−490.1
FVC (mL)	3906.6+/−822.7	2641.6+/−613.1
FEV1/FVC Ratio	0.7+/−0.1	0.8+/−0.1
Calculated mean cotinine concentration (ng/ml)	725.6+/−2731.9	436.0+/−1943.8
Emphysema (%)	21.4+/−12.3	12.8+/−9.9

**Table 5:** Cox proportional hazards models for RV measures and time to dyspnea in participants with available lung measures.

	HR*	95%CI	P value
**Right ventricle end-diastolic mass**			
Unadjusted model	1.12	1.02–1.24	0.01
Adjusted model†			
Men	1.13	0.96–1.33	0.14
Women	0.96	0.81–1.13	0.58
Adjusted model + LV end-diastolic mass			
Men	1.20	1.01–1.44	0.04
Women	1.00	0.84–1.19	1.00
**Right ventricle end-diastolic volume**			
Unadjusted model	1.10	1.03–1.17	0.004
Adjusted model†			
Men	1.15	1.02–1.30	0.02
Women	0.94	0.83–1.06	0.31
Adjusted model + LV end-diastolic volume			
Men	1.18	1.01–1.38	0.03
Women	0.96	0.82–1.13	0.63
**Right ventricle ejection fraction**			
Unadjusted model	0.93	0.86–1.01	0.06
Adjusted model†	1.01	0.93–1.10	0.75
Adjusted model + LV ejection fraction	1.00	0.91–1.10	0.99
**Right ventricle end-systolic volume**			
Unadjusted model	1.25	1.08–1.45	0.002
Adjusted model†			
Men	1.29	1.01–1.64	0.04
Women	0.80	0.60–1.05	0.10
Adjusted model + LV end-systolic volume			
Men	1.33	1.02–1.73	0.03
Women	0.82	0.61–1.10	0.17
**Right ventricle stroke volume**			
Unadjusted model	1.12	1.01–1.23	0.03
Adjusted model†			
Men	1.19	1.00–1.40	0.04
Women	0.96	0.81–1.13	0.58

* All hazard ratios correspond to ∼1 SD decrement in the respective RV measure (4 g for mass, 20 cc for volumes, 6% in RVEF).

† Adjusted model includes age, race/ethnicity, education, body mass index, waist size, hip size, smoking status, diastolic blood pressure, hypertension, creatinine, IL-6, FEV_1_, FVC, cotinine, percent emphysema, and CT scanner type.

## Discussion

We have shown that lower RV mass and smaller RV volumes in multi-ethnic community-based men without clinical cardiovascular disease at baseline were associated with an increased risk of developing dyspnea. While contrary to our original hypothesis, these findings were independent of confounders. These associations remained after adjustment for spirometric measurements, % emphysema by CT, and the respective LV parameters, suggesting that neither lung structure and function nor LV morphology account for the association of RV structure with the risk of dyspnea. RV morphology may contribute to symptoms in adults without clinical cardiovascular or pulmonary disease.

Our original hypothesis was based on what is known about RV remodeling in response to pulmonary hypertension, which results in RV hypertrophy and increasing RV volumes. The opposite results found in this study may well reflect a completely separate disease process accounting for dyspnea in these individuals free of clinical cardiovascular disease at baseline. For example, we have shown smaller LV volumes in those with subclinical chronic obstructive pulmonary disease [11], and our recent studies suggest a similar phenomenon in the RV.

We have previously shown associations between lower FEV1/FVC ratio and greater emphysema by CT and smaller LV end-diastolic volume and stroke volumes and lower cardiac output, particularly among smokers [11]. Pulmonary function could impact the RV and result in dyspnea and could certainly confound the relationship between RV morphology and dyspnea. Obstructive ventilatory physiology and emphysema, air trapping, increased intrathoracic pressure, and subsequent impaired RV filling could both shrink RV volumes and increase the risk of dyspnea. Therefore, we included available measures of spirometry, urine cotinine, and percent emphysema in multivariate analysis to “control” for lung function and emphysema. We did not find significant changes in our conclusions based on this analysis, suggesting that our findings are not due to differences in lung structure or function. Others have shown that RV failure in pulmonary hypertension is associated with reduction in LV mass [12]. Therefore, changes in RV structure and function might lead to dyspnea by affecting changes in LV structure. However, our findings persisted despite adjustment for LV measures, suggesting other explanations.

Factors affecting the intrinsic compliance or diastolic function of the RV (therefore decreasing RV volumes) may pre-dispose to dyspnea. We have previously demonstrated that being overweight or obese are independently associated with differences in RV morphology even after adjustment for the respective LV measure [13]. However, in the present study all RV parameters were adjusted for body size in multivariate analysis suggesting our results were independent of body size. In addition, we (and others) have found age-related decrements in RV mass and volumes (implying a maladaptive process) [14], [15], [16], yet our results were independent of age. RV diastolic dysfunction is a known risk factor for worse outcomes in patients with established cardiovascular disease [17], [18], but there are few data focused on RV diastolic function in large multi-ethnic populations of clinical cardiovascular disease-free adults.

There are several possible explanations for the significant associations in men but not in women. Men and women have different RV mass, volumes, and RVEF in disease-free individuals [14], [15], [19]. We postulate that our findings that lower RV mass and smaller RV volumes are associated with increased risk of developing dyspnea represents a different pathophysiologic mechanism from the one that links RV hypertrophy and increased volumes in pulmonary hypertension. Subtle RV changes could differentially impact on the sensation of dyspnea in men and women. Furthermore, the determination of incident dyspnea by questionnaire might be different for men than for women. For example, the perception of dyspnea (or response to questions) could be sex-specific. Large population-based studies suggest that the prevalence of breathlessness is greater in women than men [20]. In addition, in patients with similar severity COPD [21], asthma [22], or idiopathic pulmonary fibrosis (IPF) [23], women experience greater dyspnea than men. These sex-specific differences in reporting of dyspnea could affect the demonstrated associations with RV morphology. Alternatively, differences in RV morphology in males may have distinct implications than such differences in women. One of the strengths of MESA is the ability to detect the impact of subclinical cardiovascular changes between individuals of different sexes.

There are several potential limitations of this study. Participant report of dyspnea has inherent subjectivity. Of course, any error in the reporting of dyspnea would likely be non-differential and thus bias towards the null, so that the actual associations of RV structure and the onset of dyspnea may be even stronger than shown. In addition, interval censoring from using questionnaires administered at each face-to-face exam limited the precision in the assessment of time to dyspnea. Such censoring should again (if anything) bias to the null. There were a significant number of participants not included in our analysis given that they had dyspnea when first administered the questionnaire (Exam 2). While exclusion of these participants may decrease generalizability, inclusion of these individuals might lead to reverse causation. Last, deaths were considered as end points, since the alternative would have been to censor at death, defying the assumptions of non-informative censoring. If misclassification resulted from classifying all deaths as “dyspnea” end points (without a systematic relation to RV morphology), this would again bias to the null. Sensitivity analyses showed that our findings were robust to exclusion of death as an endpoint.

In summary, we have demonstrated an association between RV mass and volumes and the development of dyspnea in a cardiovascular disease-free population. These findings were independent of confounders and were not explained by differences in pulmonary structure and function. Significant gender interactions were present with associations between RV measures and dyspnea significant only in men. These data reveal important clinical implications of the RV in determining the risk of dyspnea and physical limitation in cardiovascular disease-free individuals in the community.
